# Lesions of the prepuce and penis in rams: A retrospective study

**DOI:** 10.1007/s11259-023-10128-8

**Published:** 2023-05-03

**Authors:** L. Falchi, S. Pau, M. Ledda, V. Melosu, MT. Zedda

**Affiliations:** Section of Obstetrics and Gynaecology, Department of Veterinary Medicine, University of Sassari, Sassari, Italy

**Keywords:** Glans penis, Urethral process, Ovine, Breeding soundness examination

## Abstract

In the ovine reproductive management, a thorough breeding soundness evaluation including specific inspection of the male genital tract represents a valuable tool for assessing the reproductive potential of a selected subject and for diagnosing genital disorders. During examination, accurate inspection on penis and prepuce is necessary, since conditions affecting these structures may hamper regular coitus. Records from 1270 males undergoing breeding soundness evaluation (n = 1232) or admitted for genital disorders to the Section of Obstetrics and Gynaecology (n = 38) of the Department of Veterinary Medicine, were collected, and lesions of penis and prepuce were therefore classified. The data collected revealed that 47/1270 rams examined presented lesions of the penis and prepuce. The most frequent condition was urolithiasis accounting for over 2% of the cases, followed by lack of the urethral process (0.39% incidence), lack of the glans penis and hypospadias (0.23% cases). Moreover, most of the conditions (40%) were observed in animals less than 2 years old, suggesting the importance of a careful breeding soundness evaluation in animals at young age.

## Introduction

Breeding soundness evaluation in rams plays an essential role in the management of sheep farming because it allows one to predict the reproductive potential of selected animals. Basically, it consists of general clinical examination, specific inspection of the reproductive tract, and semen evaluation. Although general and specific inspections alone are not able to recognize sub fertile animals, in most cases they are sufficient to detect disorders that could cause reproductive failure (Boundy 1992; Bruère and West 1993). Specific examination of the genital tract includes a careful inspection of prepuce and penis since disorders or abnormalities of these structures may hamper the coitus and fertilization and be carriers of sexually transmitted conditions.

Anatomically, the prepuce is composed of a double fold of skin. It extends from the scrotum cranially, adhering to the ventral aspect of the abdominal wall. The most cranial portion (3-4 cm long) is free and ends with the preputial orifice, commonly rounded by hair.

The penis in the ovine is a tubular fibro-elastic organ, around 40 cm long with an exiguous portion of erectile tissue. The body of the penis is characterized by a sigmoid flexure, slightly caudal to the scrotum, and the glans penis presents the urethral process in its left side (Ghoshal and Bal 1976). During ejaculation, the urethral process rotates spirally, making a wave-like movement, and sprays the semen around the external cervical os of the female genital tract (Hafez 2000).

In the literature, little can be found on the current incidence of prepuce and penis abnormalities in rams. The best-known and studied are lesions caused by infectious agents such Orf virus (Gouletsou and Fthenakis 2015), ovine herpesvirus 2 (Pritchard et al. 2008), enzootic posthitis (pizzle-rot) or ulcerative balanitis associated with Mycoplasma mycoides (Kidanemariam et al. 2005; Gocmen et al. 2019). The present retrospective study reports the incidence of pathological conditions of the penis and prepuce in Sarda rams either undergoing breeding soundness evaluation or admitted for genital disorders to the Section of Obstetrics and Gynaecology of the Department of Veterinary Medicine, University of Sassari (Sardinia- Italy), from January 2010 to December 2020.

## Materials and methods

### Criteria for selection of cases

Data were retrieved from the database of the Section of Obstetrics and Gynaecology of the Department of Veterinary Medicine of the University of Sassari (Sardinia-Italy). From January 2010 to December 2020, records from 1232 rams undergoing breeding soundness evaluation were collected. All animals were reared under traditional semi extensive systems and were examined 1 to 2 months before the beginning of the breeding season (April-May) by two operators of equal experience. Moreover, in the same years, records from 38 rams admitted for genital disorders to the Section of Obstetrics and Gynaecology of the Department of Veterinary Medicine were comprehended in the study. Each record included age, breed and identification number of the animal, date of examination, history, findings on general and genital examination, diagnosis of possible genital conditions and treatment. Lesions of penis and prepuce were included in the study.

### General clinical examination and genital inspection

A complete general examination was performed on each animal focusing on body condition score, status of limbs, feet and teeth and evidence of systemic diseases.

All males underwent careful clinical examination of the reproductive tract. The scrotum was inspected for symmetry, changes in colour of the skin, lesions, and dermatitis. The testes, epididymis, and spermatic cords were examined by palpation to assess size, shape, consistency and free movement inside the scrotum.

The internal mucosa and the external skin of the prepuce were evaluated, and a special attention was paid to the preputial orifice. The presence of signs of injuries, foreign bodies, ulcers, abnormal or blood discharge was recorded. Afterwards, examination of the penis was performed by externalization. Briefly, the ram was placed in a sitting position as vertically as possible; the sigmoid flexure was grasped firmly between the index finger and the thumb, pushing upwards towards the prepuce orifice and, at the same time, pushing the prepuce skin downwards. When the penis was extruded, particular attention was paid to any difficulty in the passage through the preputial orifice. The glans and the urethral process were assessed for alterations (wounds, ulceration, inflammation, necrosis, absence). Any abnormal condition of prepuce and penis was recorded and described.

## Results

Lesions of the penis and prepuce were recorded on 47 out of 1270 rams (3.7% incidence; Table 1). The age distribution of examined rams ranged between < 1 year to 6 years and it is summarised in Table 2. Multiple abnormalities were considered as singular cases.

Three rams less than one year of age, from three different farms, were diagnosed with hypospadias. They presented a urethra opening on the ventral aspect of the penis, absence of the urethral process, and a shorter prepuce. In two subjects, a split scrotum was observed.

Conditions affecting the prepuce accounted for only 0.23% (3/1270) of total cases. An older ram of 5–6 years of age, undergoing a routine breeding soundness evaluation, was diagnosed with a preputial papilloma. Papillomas were found also in the scrotum, in the lower left eyelid and in the skin of the back. Another two rams were admitted to the Section of Obstetrics for an abscess and a traumatic injury. In the first case, the prepuce appeared swollen, hot, and painful and penis exteriorization was not possible. Bacterial isolation was not performed. The traumatic injury was characterised by a laceration involving all layers of the skin of the preputial orifice.

Injuries of the glans penis have been found in two animals. One was an injury caused by a penetrating foreign body on the dorsal aspect of the glans while the other was a laceration.

**Fig. 1:**
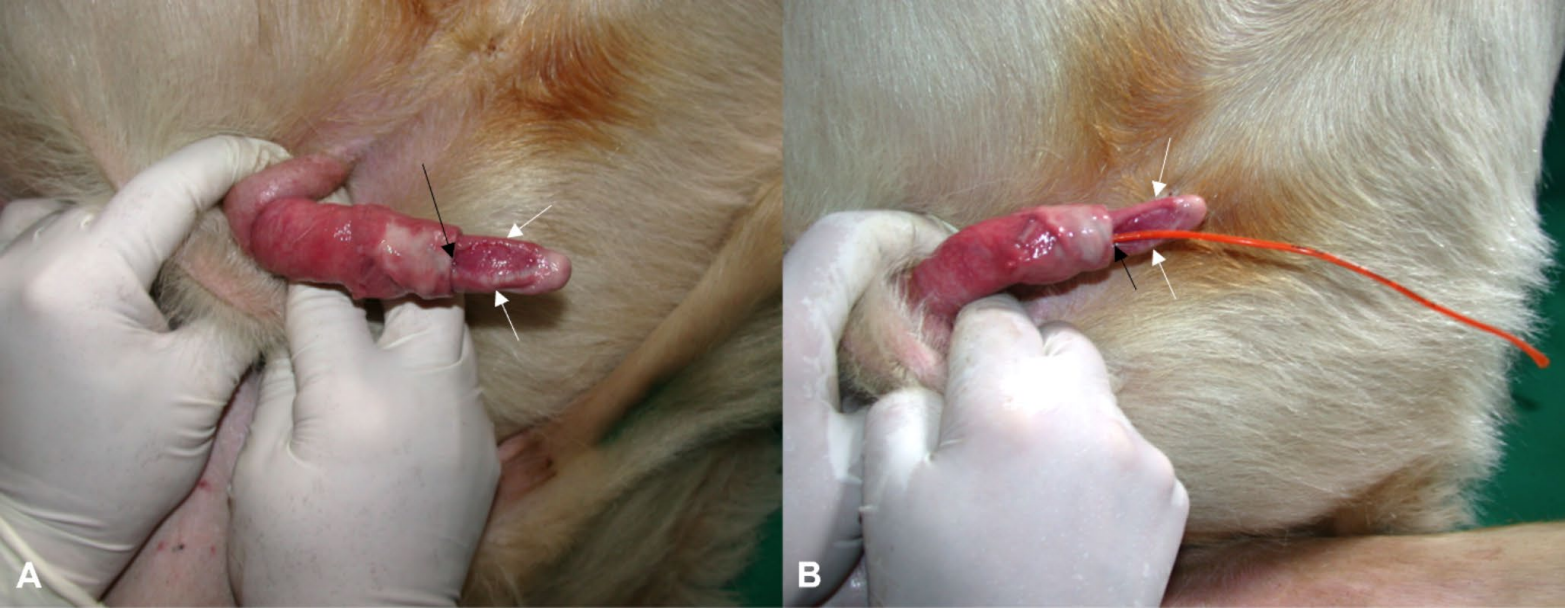
Complete absence of the glans penis in a 4-year-old ram. (**A**) The distal part of the penis presented, on the ventral aspect, a groove, delimited by edges (white arrows), containing caudally the urethral opening (black arrow); (**B**) the urethral opening was pointed out by the insertion of a urinary catheter

A ram of 3–4 years of age, undergoing a routine breeding soundness evaluation, was diagnosed with the complete absence of the glans penis. In the anamnesis, no data regarding the mating efficiency and fertility were provided. During examination, complete exteriorisation of the penis was not possible due to adhesions. The apical portion of the penis was rounded and characterised by tight tissue. On its ventral aspect, it presented a groove containing the urethral opening, as pointed out by the insertion of a urinary catheter (Fig. 1).

**Fig. 2:**
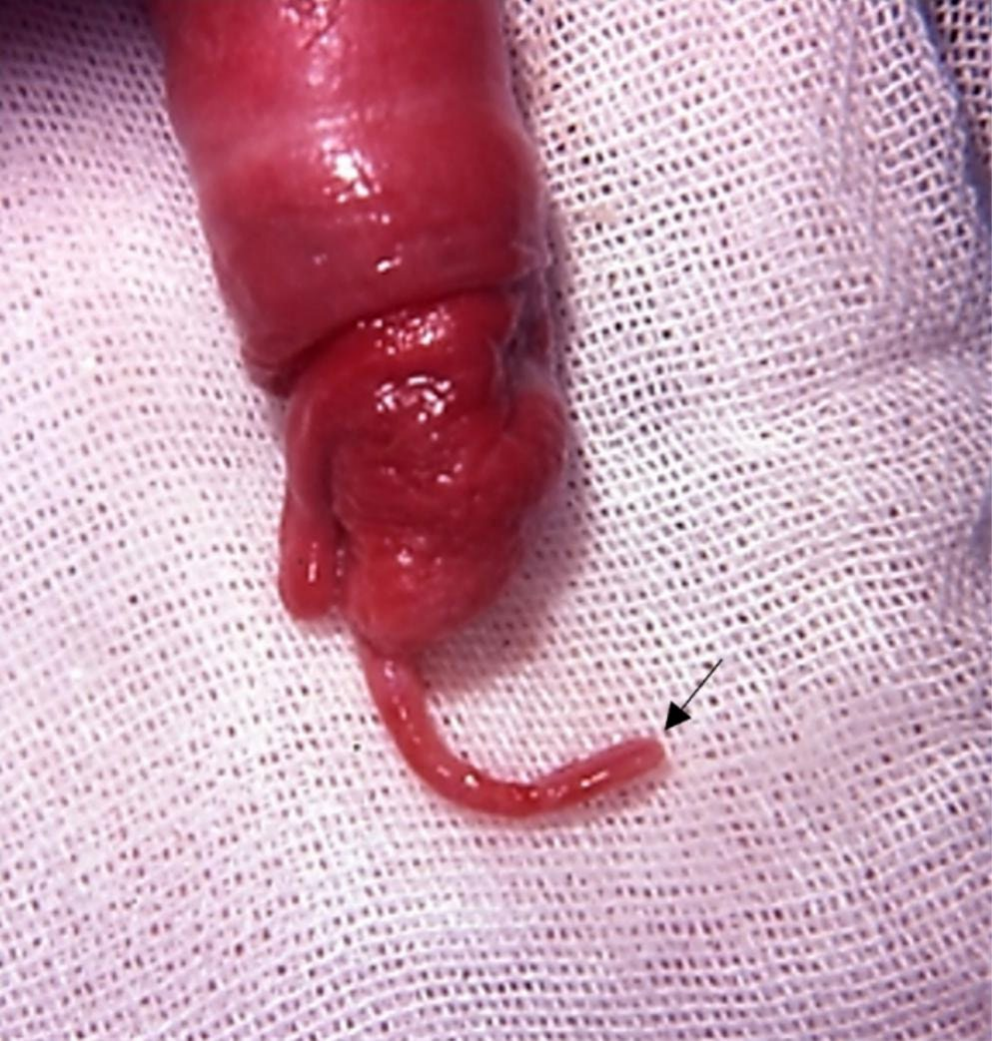
Retroflexion of the urethral process in a 3-year-old ram. Adhesions kept the apical part of the process bent at around 1 cm from the tip (black arrow)

The absence of the urethral process was observed in 5/1270 cases (0.39% incidence). The restraint of the animal during shearing with consequent accidental amputation of the process (as reported by the owners) was the cause in 3 out of 5 cases). In one subject, retroflexion of the urethral process was described. Despite a normal libido, the ram was hypofertile, reluctant to mount and had urine retention in the preputial cavity. Following examination, a balanoposthitis and a completely bent urethral process were observed. The urethral process appeared retroflexed at around 1 cm from the tip, and adhesions kept the bent portion attached to the main part of the process (Fig. 2).

Urolithiasis accounted for over 2% of the cases (28/1270). All rams were admitted to our section with an history of inappetence, stranguria and ematuria. Three of them also presented light preputial swelling. Eight subjects affected by urinary obstruction also presented pressure necrosis of the urethral process and penis at the presumptive obstruction site and downstream (8/1270; 0.63% incidence) Urolithiasis affected more frequently young animals: 23/28 were less than 2 years old. On the 28 cases, 16 males, including those affected by necrosis of the penis, were euthanised because owners declined treatment and only four of them gave consent for necropsy. Uroliths were found in the bladder (4/4), in the sigmoid flexure (3/4), in the penile urethra (2/4) and in the urethral process (3/4) and composition analyses revealed all uroliths to be composed of struvite.

**Table 1:** Age distribution of ram lambs and adults examined by routinary breeding soundness evaluation or admitted to the Department of Veterinary Medicine for disorders of the genital tract

*Age distribution of rams*		
*Age*	*Number*	*%*
*≤ 1 year*	337	26.5
*1–2 years*	260	20.5
*3–4 years*	483	38
*5–6 years*	173	13.6
*> 6*	17	1.3
*Total*	1270	

**Table 2:** Distribution and incidence of lesions of penis and prepuce and number of affected animals according to the age. Incidences were calculated on the total number of examined rams (n = 1270). * Retroflexion of the urethral process and balanoposthitis were observed in the same subject

*Abnormalities*	*Number of cases*	*Incidence* *(%)*	*Age (years)*			
			*< 1*	*1–2*	*3–4*	*5–6*
*Hypospadias*	3	0.21	3			
*Preputial abscess*	1	0.07		1		
*Preputial papilloma*	1	0.07				1
*Injuries of the preputial skin*	1	0.07				1
*Urolithiasis*						
*With necrosis of the penis and urethral process*	8	0.62	3	1	2	2
*Without necrosis of the penis*	20	1.57	13	6		1
*Injuries of the glans penis*	2	0.16		1		1
*Lack of the glans penis*	1	0.07			1	
Retroflexion of the urethral process*	1	0.07			1	
Balanoposthitis*	1	0.07			1	
*Lack of the urethral process*	5	0.39		2	3	
*Phimosis*	1	0.07				1
**TOTAL**	**47**	**3.7**	**19**	**11**	**8**	**7**

## Discussion

The majority of conditions affecting the penis and the prepuce can be easily detected by an accurate clinical examination.

Among the congenital disorders of the penis and prepuce, the only one reported in this study was hypospadias, affecting three young rams of less than 1 year old. In two of them a split scrotum was also present. These are not an uncommon finding, since hypospadias is often associated with other abnormalities of the reproductive tract (Smith et al. 2012). Hypospadias is a congenital disorder that consists in a defect in the closure of the urethral grooves and in the opening of the urethra at some point of the ventral aspect of either the glans, penis, scrotum, perineum, or anus (Smith et al. 2012). Although this condition is one of the most common in humans (four to six males/1000), as reviewed by Carmichael et al. (2012), it is not frequently reported in domestic animals. In Southwest England, 15 cases of hypospadias were reported (incidence 0.21%) in an observational study carried out on 7307 rams, (Smith et al. 2012). A similar incidence, despite the limited number of animals observed, was found in the present report (3/1270; 0.23%). Although in literature little can be found on the heritability of this condition, this component cannot be ruled out (Bleul et al. 2007; Iannuzzi et al. 2020) and young lambs with hypospadias are commonly removed from the flock being unfit for mating. The three lambs diagnosed with hypospadias belonged to three different owners and therefore we cannot speculate about heritability being a predisposing factor.

In our report, injuries of the prepuce and the penis accounted for 0.3% of total animal examined. This incidence was surprisingly low compared with what found in other species. In commercial farms of boars, Weiler et al. (2016) reported a striking higher incidence, ranging from 64 to 94.9% (Weiler et al. 2016) with around 9.3% of them showing severe lesions. In bulls, lesions and scars in the penis and prepuce accounted for 6% and 15% respectively of all conditions affecting the penis and prepuce in a population of over 2000 animals (Queirolo 1983). We can speculate that the lower occurrence found in our study may be explained considering the economic cost of treatment weighed against the ram’s replacement value, that leads the owner of the animal to prefer euthanasia rather than a therapeutic approach.

A single case of neoplasm was reported in our study, a preputial papilloma in a ram of 5 to 6 years of age. Immunohistochemistry and genome isolation were performed to isolate a deltapapillomavirus, the etiological agent. To our knowledge, in small ruminants, no case reports are available on these neoplasms of the penis and prepuce, while in the horse and the bull penile or preputial warts have been often described (Gardiner et al. 2008; Wolfe 2018). This condition is mainly detected during an accurate breeding soundness evaluation or semen collection (Wolfe 2018).

An interesting finding in this retrospective study regarded the complete lack of the urethral process observed in 5 subjects, caused in most of the cases, by incorrect restraint of the animal during shearing, as also previously reported by Bruère and West (1993). This condition was accidentally detected during routine breeding soundness evaluation. According to our experience, the lack of urethral process should not affect the reproductive soundness and the mating ability of the male. In support of this observation, Thomas et al. (1990) reported no alteration in the volume of ejaculates, concentration, morphology and motility of spermatozoa in rams experimentally undergoing urethral process removal on semen quality. In contrast, some authors suggest that the urethral process should be preserved since its removal may lead to hypo- or infertility (Boundy 1992).

The most common condition observed in this retrospective study was urolithiasis, accounting for over 2% of the cases (28/1270) affecting more frequently young animals (less than 2 years old of age). This was not surprising since age is considered a risk factor due to the smaller diameter of the urethral lumen (Riedi et al. 2018; Cook 2023). Urolithiasis is a multifactorial clinical emergency and a significant cause of morbidity and mortality. Besides age, other predisposing factors such as diet, urine pH and dehydration play a significant role in the aetiology of this condition (Hay 1990). The analysis of uroliths helps in understanding the cause of mineral deposition (Jones et al. 2017). Struvite and apatite crystals are commonly found in animals fed with high grain diets, calcium crystal are often related to rations high in legumes and pelleted diets predispose to the formation of phosphorous crystals. In our report, all cases in which uroliths analysis was performed revealed a composition of struvite crystals. After a careful anamnesis, a high grain diet and pasture on alcalyne soils were considered to be the predisposing factors (Jones et al. 2017). Uroliths may obstruct the regular flow of urine anywhere along the urinary tract but, being the urethra in small ruminants long, narrow, and tortuous, the most frequent obstruction sites are the sigmoid flexure and the urethral process (Tiruneh 2000; Tibary and Metre 2004). Partial or total obstruction of the flow may lead to inappetence, restlessness, dysuria and stranguria in less severe cases, and pain, ascites, and ventral abdominal swelling in cases in which rupture of the bladder or urethra occur (Riedi et al. 2018).

In the present retrospective study, animals younger than 1 year represent 26.5% of the investigated population. In the same age group, 40.4% percent of all lesions were found. These data strongly suggests that an early breeding soundness evaluation, performed in pubertal animals, would support the optimization of selection programs, leading to early diagnosis and treatment of genital diseases or replacement of the subject.

## Data Availability

The datasets analysed during the current study are available from the corresponding author on reasonable request.
